# The role of obstructive sleep apnea, neurofilaments and early CPAP intervention in post-stroke cognitive recovery

**DOI:** 10.1016/j.sleepx.2025.100142

**Published:** 2025-05-31

**Authors:** Petra Levicka, Miriam Slavkovska, Dominik Koren, Joaquim Ventosa, Ján Hlodak, Jana Papikova, Zuzana Gdovinova, Eva Feketeova

**Affiliations:** a Univerzita Pavla Jozefa Safarika V Kosiciach, Lekarska Fakulta, Neurologicka Klinika, Slovakia; b Univerzitna Nemocnica L. Pasteura, Kosice, Slovakia; c Univerzita Komenskeho V Bratislave Fakulta Socialnych a Ekonomickych Vied, Bratislava, Slovakia

**Keywords:** Cognitive decline, Post-stroke outcome, Obstructive sleep apnea, Neurodegeneration biomarker, Neurofilament light chain, Adherence to CPAP treatment

## Abstract

Stroke is a leading cause of disability worldwide, with cognitive impairment following stroke influenced by a complex interplay of modifiable and non-modifiable risk factors.

This study investigated the impact of obstructive sleep apnea (OSA) on cognitive outcomes after ischemic stroke (IS) and the predictive value of plasma neurofilament light chain (pNFL) levels. Seventy-three acute IS patients were analyzed, with 59 completing a three-month follow-up. Cognitive function (Montreal Cognitive Assessment, MoCA) was assessed. Patients underwent polygraphic screening for OSA in the acute phase, with treatment recommended when indicated, and pNFL levels measured at baseline and follow-up.

Results showed that 93.2 % of IS patients had OSA. Forty (72.7 %) of OSA patients (moderate, severe OSA) were recommended continuous positive airway pressure (CPAP). CPAP-treated patients in the acute phase demonstrated cognitive improvement at three-month follow-up (CPAP-treated: MoCA 23 vs 25 points, CPAP indicated untreated, MoCA 22 vs 22 points, p = 0.05). However, long-term adherence to CPAP was poor - only 25 % remained on therapy at three months. While pNFL levels correlated with infarct volume and significantly decreased over time, no correlation was found between OSA severity and CPAP treatment. Regression analysis identified age, prior stroke history, and anxiety as key predictors of cognitive and functional post-stroke outcome.

Early CPAP therapy could contribute to improved post-stroke cognitive performance. Decline in pNFL levels shows ongoing neuronal recovery; a direct relationship with OSA is inconclusive. Furthermore, advanced age, history of prior stroke, and anxiety symptoms emerged as significant contributors to poorer cognitive outcomes.

## Highlights

• Early CPAP treatment in stroke patients could improve further cognitive performance.
• Neurofilament light Chains show ongoing neuronal recovery in stroke.
• Changes in Neurofilament light Chains and OSA and its treatment remain inconclusive.
• Age, prior stroke, and anxiety symptoms contribute to poorer cognitive outcome.

## Graphical abstract

**Figure:**
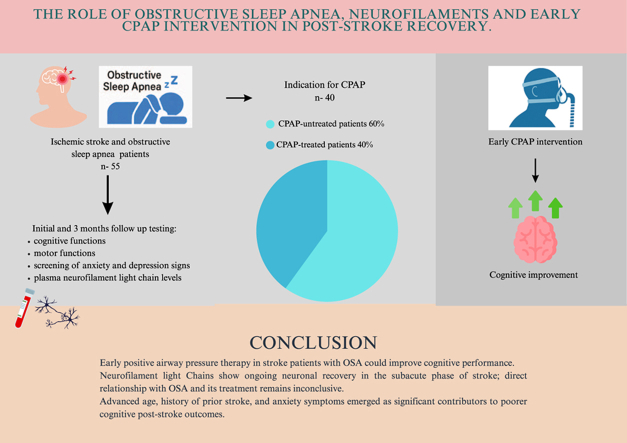

## 1 Introduction

Stroke is one of the most common causes of disability in Slovakia and worldwide. Thanks to continuous advances in the management of acute treatment, the functional status of these patients is improving. However, post-stroke cognitive impairment (PSCI) and dementia after stroke are still highly prevalent and disabling [1] despite state-of-the-art stroke treatment [2], which calls for new interventions).

OSA is among the risk factors of stroke and cardiovascular disorders, which have been recognized in recent decades [3]. High prevalence of OSA was found in cerebrovascular disease [4]. OSA increases the risk of stroke recurrence, may increase long-term mortality [[5], [6], [7]], and its severity may affect functional recovery after stroke [8].

OSA itself could participate in cognitive decline by neuropsychological effects, including vigilance, attention, memory, and executive function, typically present in younger middle-aged adults. It includes daytime sleepiness due to fragmented sleep because of frequent respiratory events [9]. Older adults show a decline in a global measure of cognition, with a modest association between nocturnal hypoxemia and subsequent decline [10]. Cognitive outcome after stroke requires complex evaluation for other sources of decreased cognitive performance. Unrecognized Alzheimer's disease (AD) may contribute to cognitive deficit [11]. Even more vascular factors predisposing to stroke and cerebrovascular disease have been associated with dementia and probably more specifically with the enhancement of amyloid-β (Aβ) deposition [12]. The blood-brain barrier, perivascular space, and the glymphatic system, the latter proposedly responsible for the drainage of solutes from the brain parenchyma, may represent key pathophysiological pathways linking stroke, Aβ deposition, and dementia [13] and OSA [14,15]. Much attention is currently devoted to uncovering PSCI risk factors and their biomarkers. NFL levels in cerebrospinal fluid and blood can differentiate individuals with AD from healthy controls with moderate accuracy (Area Under the Curve ∼.7). However, the NFL is associated with neurodegeneration, mainly white matter damage, rather than amyloid pathology, making it less specific for AD, as it increases in various neurodegenerative diseases [16].

The size, number and location of the stroke can predict post-stroke cognitive deficit [17,18]. Cognitive reserve as years of education independently protects post-stroke cognition [2].

Currently, in addition to the treatment of the stroke, we have OSA treatment available as the only other treatable factor involved in PSCI. While OSA treatment, particularly CPAP therapy, is known to improve sleep quality and reduce cardiovascular risks, evidence on its impact on post-stroke functional outcomes is limited and still emerging. A meta-analysis evaluated the impact of CPAP therapy on cognitive function in stroke patients with OSA. Analysing seven randomized controlled trials with 327 participants, the study found that CPAP treatment did not significantly improve global cognitive function overall. A subgroup analysis (70 participants) revealed that initiating CPAP therapy within two weeks post-stroke significantly enhanced global cognition and reduced subjective sleepiness [19].

Despite the increasing information about OSA and IS, it is not clear from studies to date how and whether patients with IS benefit from early diagnosis and treatment of OSA.

We hypothesized that.

1. PNFL levels can predict cognitive outcome in stroke survivors, and the impact of OSA on neuronal damage.
2. Positive airway pressure therapy in IS patients with moderate/severe OSA will be associated with more prominent decrease of pNFL levels and could prevent further cognitive decline.

## 2 Materials and methods

**Fig. 1:**
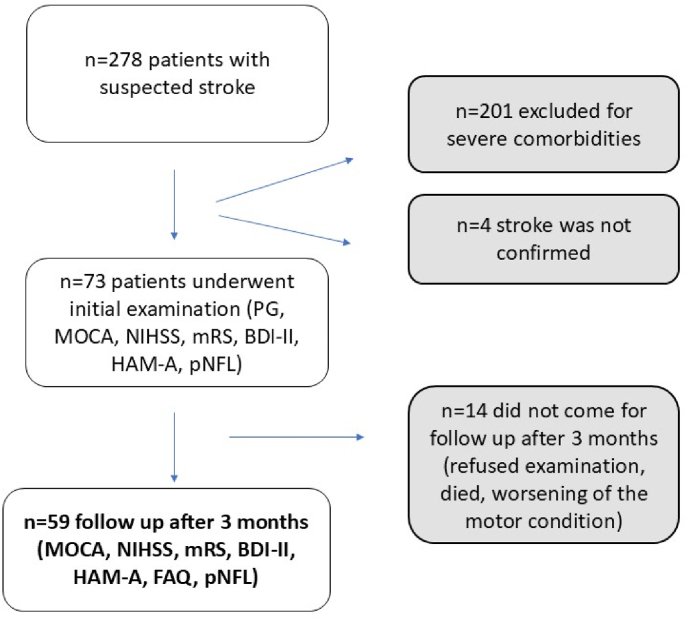
Flowchart-it illustrates the selection and follow-up of patients with suspected stroke. *Abbreviation (Legend):* PG-polygraphic screening, MoCA – Montreal cognitive assessment, BDI II– Beck depression inventory, HAM-A Hamilton Anxiety Rating scale, NIHSS – National institutes of health stroke scale, mRS – modified Rankin scale, pNFL - plasma neurofilament light chain, FAQ – Functional Activities Questionnaire.

A total of 278 patients in the acute stage of IS without specific age restriction were included in the study over 2 years (2022–2024). Two hundred and five patients were ultimately excluded from the study because they could not cooperate. These were predominantly patients with delirium, prefrontal syndrome, severe neurological deficit (National institutes of health stroke scale, NIHSS >20 points), or severe heart and lung disease. We excluded four patients, as the diagnosis of stroke was not confirmed in their further course (it was a brain abscess, brain metastases, and Todd's paresis after an epileptic seizure). In the end, 73 patients were available for our analysis, of which 59 participated in the follow-up examination (see Fig. 1).

Data collection included age, sex, height and weight, based on which we calculated body mass index (BMI), personal history (presence of arterial hypertension, diabetes mellitus, dyslipidemia, heart disease, arrhythmias, history of previous stroke), smoking and alcohol abuse history and the result of brain imaging examinations including the information of infarct volume based on magnetic resonance imaging.

All participants signed the informed consent. This study was approved by the Ethics Committee of Louis Pasteur University Hospital in Kosice, Slovakia. Data were collected in compliance with the ethical principles outlined in the 1964 Declaration of Helsinki. Each subject who fulfilled the selection criteria underwent a cardiorespiratory polygraphic screening (PG) to detect sleep-disordered breathing.

We used polygraphs Samoa (Löwenstein, Germany) and Porti 8 (Philips Respironics, USA) for sleep studies. Respiration (oronasal transducer), respiratory effort (Thorax/Abdomen pressure pads), body position, SpO2, heart rate derived from oximeter were recorded. Raw data were manually scored by sleep fellow (PL) and somnologist (EF) and expressed as monitoring time, respiratory events index (REI)- hypopnea and apnea per hour of monitoring time, respiratory events were differenciated as central, obstructive and mixed), oxygen desaturation index ≥3 %, according to the AASM Manual for the Scoring of Sleep and Associated Events: Rules, Terminology and Technical Specifications, Version 3 [20]. REI surrogated Apnea hypopnea index (AHI) used in polysomnography scoring. Based on results, we diagnosed patients with mild (REI 5–15/h of sleep), moderate (REI 15–30/h), and severe (REI above 30/h) OSA. If the presence of OSA with REI >15 was confirmed, the patient was offered treatment with CPAP.

In the acute stage of IS and at the 3-month follow-up, we performed cognitive testing using MoCA scale; patients filled out Hamilton Anxiety Rating scale (HAM-A) and Beck depression inventory (BDI). Motor function was assessed using NIHSS and the modified Rankin Scale (mRS) on the last day of hospitalization and 3 months later. At the 3-month follow-up, we also investigated the impact of stroke and cognitive performance deficit on the implementation of everyday daily activities using Functional Activities Questionnaire (FAQ), with the participation of the patients relatives to evaluating the change compared to the state before the stroke.

Our protocol also included a laboratory examination of a blood sample to determine pNFL. Samples for pNFL evaluation were processed as follows: 3 mL of blood were collected from patients and kept at room temperature for 120 min and then centrifuged at 4000 rpm. during 10 min. We stored the collected plasma samples in a polypropylene tube at a temperature of −80 °C. PNFL concentrations were analyzed using SIMOATM (Single Molecule Array, NF-Light Advantage Kit, SIMOA HD-1 analyzer, Quanterix protocol, Lexington, MA, USA). In each patient, pNFL was measured at two time intervals - 5–10 days after stroke and at a 3-month follow-up.

### 2.1 Data analysis

The data were processed in a Microsoft Excel table, then transferred and statistically analyzed using IBM SPSS software version 29 for MAC. Raw scores were calculated for each scale. Descriptive statistics were conducted to describe the sample, focusing on frequency distribution, percentages, minimum and maximum values, means and medians, standard deviation, and missing data analysis. To assess data normality, the Kolmogorov-Smirnov and Shapiro-Wilk tests were performed, and based on skewness and kurtosis values, the data were evaluated as non-normally distributed. Given the small sample size, non-parametric statistical analyses were subsequently conducted. For difference testing, the Wilcoxon Signed Rank test was used for dependent samples when comparing pre- and post-intervention measurements. Mann-Whitney *U* test was calculated to observe between-group differences. Significance values were assessed at the 95 % confidence level. To examine correlations between variables, the Biserial Correlation Analysis method was applied, as the indicators of risk factors were recorded as dichotomous variables. To assess relationships between risk factors and measured indicators of cognitive and functional status in patients, a multiple regression analysis was conducted using the stepwise method.

## 3 Results

Table 1 presents the basic demographic and clinical characteristics of the study cohort.

**Table 1:** Demographic and clinical data of the patients with ischemic stroke.

Patients with ischemic stroke	n = 59	
**Gender (n,%)**	**men**	34 (57.6 %)
	**women**	25 (42.4 %)
**Age (mean ± SD years)**		68.5 ± 10.7
**Risk factors (n,% or mean ± SD)**	**Arterial hypertension**	52 (88.1 %)
	**Atrial fibrillation**	3 (5.1 %)
	**Diabetes mellitus Type 2**	15 (25.4 %)
	**Hyperlipidemia**	40 (67.8 %)
	**Atherosclerosis**	50 (84.7 %)
	**History of ischemic stroke**	9 (15.6 %)
	**History of myocardial infarction**	8 (13.5 %)
	**Smoking**	16 (27.1 %)
	**Obstructive sleep apnea**	
	Not present	4 (6.7 %)
	mild	15 (25.4 %)
	moderate	15 (25.4 %)
	severe	25 (42.4 %)
	**Body mass index** (kg/m2)	27.91 ± 2.72
**Antithrombotic drugs before stroke (n,%)**	**antiplatelet agents**	19 (32.2 %)
	**anticoagulants**	1 (1.7 %)
**Acute reperfusion treatment (n,%)**	**IVT**	5 (8.5 %)
	**EVT**	5(8.5 %)
	**IVT + EVT**	2 (3.4 %)
**Etiology of stroke (n,%)**	**cardioembolic**	6 (10.1 %)
	**atherosclerotic**	27 (45.8 %)
	**lacunar**	10 (16.9 %)
	**cryptogenic**	15 (25.4 %)
**Infarct volume (mean ± SD ml)**	46.46 ± 57.45	

Legend: IVT - Intravenous thrombolysis, rTPA – recombinant Tissue Plasminogen Activator, EVT - Endovascular treatment.

PG screening identified OSA in 55 (93.3 %) patients. The number of the central respiratory events did not exceed 50 % in any patient, 25 patients (45 %) with moderate or severe OSA (none from mild groupe) revealed Cheyne-Stokes breathing on polygraphy. Fourty (72.7 %) patients (moderate-to-severe OSA) met the criteria for CPAP therapy. CPAP treatment was initiated the night following the diagnostic assessment, and adherence was monitored over a 10–14-day hospitalization period. Sixteen patients (40 %) agreed to the proposed CPAP therapy. Patients, family members, and caregivers received detailed instructions on home device usage before hospital discharge. However, at the three-month follow-up, only four (25 %) of patients prescribed CPAP therapy maintained adherence, defined as usage for at least 4 h per night on at least five nights per week.

Cognitive, motor, and psychosocial functions were assessed in the acute phase of IS and re-evaluated at the three-month follow-up. Cognitive performance, measured by MoCA, improved in 64.4 % of patients, whereas cognitive decline was observed in 28.8 %.

Motor function, evaluated using the mRS, improved in 35.6 % of patients, worsened in 15.3 %, and remained unchanged in 49.1 %. Additionally, as assessed by FAQ, one-third of patients experienced a decline in their ability to perform daily activities compared to their pre-stroke status.

PNFL levels were measured in the acute phase and at the three-month follow-up, revealing a significant reduction over time (Table 2). PNFL levels in the acute phase strongly correlated with infarct volume. However, no correlation was found between OSA severity (measured by REI or desaturation index) and pNFL levels at either time point.

**Table 2:** Cognitive, motor and psychosocial measures of ischemic stroke (IS) evaluated in the subacute phase of IS (T0) and 3 months later (T1).

		T0	T1	Wilcoxon test (Z)	p
**MoCA score**	**mean, SD**	22.46 ± 4.03	23.52 ± 3.86	−2.52	.012
	**without cognitive impairment (N, %)**	15 (25.4 %)	21 (35.6 %)		
	**mild cognitive impairment (N, %)**	37 (62.7 %)	34 (57.6 %)		
	**moderate cognitive impairment (N, %)**	7 (11.9 %)	4 (6.7 %)		
**Depression – BDI-II**	**mean, SD**	3.04	3.46	−1.56	.118
	**minimal signs (N, %)**	58 (98.3 %)	55 (93.2 %)		
	**mild signs (N, %)**	1 (1.7 %)	4 (6.8 %)		
**Anxiety – HAM-A**	**mean, SD**	10.44	10.93	−1.31	.189
	**normal to mild (N, %)**	44 (74.6 %)	43 (72.8 %)		
	**moderate to severe (N, %)**	15 (25.4 %)	16 (27.1 %)		
**FAQ**	**Deterioration of functional abilities (N, %)**	N/A	18 (30.5 %)		
**NIHSS score**	**mean, SD**	2.02 ± 1.58	1.05 ± 1.17	−4.94	<.001
**mRS score**	**mean, SD**	1.17 ± 1.18	.83 ± 1.18	−2.27	.023
**pNFL (pg/ml)**	**mean, SD**	266.50 ± 778.68	37.47 ± 36.07	−5.11	<.01

*Legend*: MoCA – Montreal cognitive assessment, BDI – Beck depression inventory, HAM-A Hamilton anxiety rankin scale, FAQ – Functional Activities Questionnaire, NIHSS – National institutes of health stroke scale, mRS – modified Rankin scale, pNFL - plasma neurofilament light chain.

When comparing patients eligible for CPAP therapy (moderate-to-severe OSA), no significant difference was observed in pNFL reduction between those who adhered to CPAP and those who declined treatment. However, patients who received CPAP demonstrated a statistically significant improvement (p = 0.011) in cognitive performance (MoCA) at the three-month follow-up. We noted a trend towards an improvement in the average MoCA score compared to the first examination by almost 2 points of raw score on average (23.13 vs 25.38 points) in the group of patients indicated and treated with CPAP, with a borderline significance result of p = 0.05. In contrast, in the group of untreated and indicated patients for CPAP, the mean values of MoCA did not change in the three-month follow-up on average (22.06 vs 22.04 points) and therefore were not significantly different.

Improvements were also observed in NIHSS and mRS scores, but these were not statistically significant (NIHSS: p = 0.52; mRS: p = 0.14).

Regression analysis was conducted to determine the influence of risk factors on cognitive and motor function outcomes at three months post-stroke. The model explained 40 % of the variance in MoCA scores (26.4 % after adjustment for independent variables). After removing the least significant variables (arterial hypertension, atrial fibrillation, myocardial infarction, depression, OSA, and smoking), the model retained statistical significance (p < 0.001), explaining 39.5 % of the variance (33.7 % after adjustment). The most significant predictors of cognitive performance were previous stroke, anxiety, and age, all of which negatively impacted outcomes (see Table 3).

**Table 3:** Stroke risk factors and their impact on cognitive performance by MoCA (Montreal cognitive assessment).

Coefficients	B	t	Sig.	Model R	R^2^	F	95 % CI	Sig.
Constant	35.487	12.611	<.001	.584	.341	9.494	29.84–41.12	<.001
Previous Stroke	−3.94	−3.223	.002					
Anxiety	−.161	−2.557	.013					
Age	−.143	−3.592	<.001					

Regression models for mRS and NIHSS did not yield significant results. However, regression analysis for FAQ indicated that, after excluding non-significant variables (arterial hypertension, atrial fibrillation, myocardial infarction, smoking, and depression), the model accounted for 25.6 % of variance (33.5 % without adjustment). The same key predictors: age (p = 0.008), previous stroke (p = 0.027), and anxiety (p = 0.005) were identified as the strongest determinants of functional decline in daily activities.

## 4 Discussion

The presented study was conducted to analyze the impact of risk factors, including sleep apnea and plasma biomarker NFL levels, on the prediction of cognitive and motor outcomes after the IS.

### 4.1 Stroke and OSA, CPAP treatment, and stroke outcomes

Several studies have proven OSA to represent an independent risk factor for IS. Sleep studies in stroke survivors are recommended because of the high prevalence of OSA after stroke, which is between 50 % and 70 %, not depending on event type or type of monitoring [21,22]. In our sample, over 90 % were diagnosed with OSA in the acute phase after IS, a prevalence higher than typically reported. It is possible that the high prevalence of OSA in our sample was related to the timing of the examination in the acute phase of stroke, with acute hemodynamic changes in the early stage of neuronal damage potentially influencing the results. However, the meta-analysis on the prevalence of sleep-disordered breathing after stroke did not show a variety across the acute to the subacute and chronic stages [23]. The BMI in the presented sample did not exceed the normal range for the age group and could not explain the OSA higher prevalence.

More than two-thirds of our patients with OSA were recommended CPAP therapy, with 40 % consenting treatment, only 25 % of them were adherent to the treatment after a three-month follow-up, what did not allow us to evaluate the effect of long-term treatment intervention on stroke outcomes or pNFL levels. Notably, preliminary findings indicate that early CPAP use initiated and controlled during hospitalization (mean 14 days, even later temporary), led to an improvement in cognitive and motor performance at the 3-month follow-up compared to patients with OSA who initially refused CPAP treatment. A meta-analysis by Yang et al. [19] reported similar conclusions. Caution is needed when interpreting the findings, as studies have also admitted spontaneous improvement of post-stroke cognitive functions in some patients [24,25].

Excessive daytime sleepiness (EDS), one of the major symptoms of OSA, has been suggested as a factor motivating CPAP adherence, given the improvement in EDS with CPAP therapy [26]. Thus, individuals with OSA but without EDS may perceive a lack of benefit that mitigates CPAP adherence. In the RICCADSA trial, the probability of remaining on CPAP at 2 years was 60 % in nonsleepy patients compared to 77 % in patients with EDS at baseline [27]. None of our 16 patients, who agreed with CPAP therapy, exhibited EDS, which we assume may be related to the low long-term adherence to treatment. Additional barriers may have included insufficient family support, poor interdisciplinary collaboration, and limited awareness of CPAP as a rehabilitation tool within the country's healthcare settings. Furthermore, none of the 278 stroke patients included in our study had been previously diagnosed or treated for OSA prior to IS, which may reflect systemic limitations in screening and diagnostic capacity in Slovakia.

Patients with severe OSA are generally thought to derive the most benefit from CPAP therapy and may exhibit better adherence. However, existing data do not clarify whether patients with poor adherence due to neurological deficits had more severe OSA. We can only speculate that these individuals may have had greater potential for recovery with appropriate intervention. Further research on unselected stroke cohorts is essential to better understand these associations.

### 4.2 pNFL, OSA, post-stroke cognitive performance

In the group of our patients, pNFL values significantly decreased in a 3-month follow-up. They correlated with the volume of the ischemic lesion and did not correlate with cognitive performance. We assume the pNFL levels in the presented study served as a marker of acute neuronal injury rather than predictor of cognitive or functional stroke outcomes. However the study of Jiang et al. demonstrated that higher NFL concentrations in post-stroke patients correlated with cognitive deterioration over a 12-month period [28].

We hypothesized that in patients with moderate and severe OSA who will be treated with CPAP there will be a more significant decrease in the level of pNFL at a three-month follow-up compared to patients with OSA who refused the treatment. The results did not show statistical significance between the compared groups of patients.

A longitudinal study by Sehr et al. investigated the effects of OSA treatment on neurodegenerative biomarkers, revealing that elevated serum NFL levels were associated with cognitive impairment in OSA patients. The study demonstrated that effective OSA treatment with CPAP therapy not only enhanced cognitive performance but also reduced serum NFL levels, indicating reduced neuronal damage [29]. However, no other study has been conducted evaluating the coincidence of IS and OSA on PSCI and motor stroke outcomes and pNFL levels.

### 4.3 Other predictors of stroke outcomes

Cognitive and motor performance showed a trend to improve during the three-month follow-up after IS, but poorer outcomes were associated with older age, apparently a smaller functional reserve with a history of IS overcome, and the influence of anxiety symptoms. Age-related differences in recovery are well-documented, with patients over 70 experiencing limited functional gains. While younger individuals show improvements in motor function and daily activities for up to six months, older patients often plateau within the first month [30]. Similarly, cognitive recovery appears age-dependent, with younger patients improving for six months, whereas older individuals tend to stagnate early, underscoring the need for targeted cognitive rehabilitation [31].

Recurrent strokes lead to cumulative neurological damage, increasing the risk of cognitive impairment, which affects up to 60 % of survivors within a year. Studies show that 37 % of stroke and transient ischemic attack survivors develop mild cognitive impairment, while 22 % progress to dementia over seven years [32]. Functional dependence is a frequent outcome, with cognitive deficits predicting long-term dependence and reduced quality of life [33]. Prior strokes are also a strong predictor of post-stroke dementia [34]. Anxiety further exacerbates recovery challenges, as it hinders rehabilitation, reduces participation in recovery programs, and negatively affects daily activities. Post-stroke anxiety has been linked to poor long-term functional outcomes [35], and its co-occurrence with depression may further impair cognitive recovery and quality of life [36].

## 5 Strengths and limitations

Strengths of this study include the comprehensive evaluation of the factors contributing to post-stroke cognitive performance, including OSA and the effects of early OSA intervention, neurodegeneration (via pNFL), depressive and anxiety symptoms, cardiocerebrovascular burden and age.

However, the study is limited by its sample size and the absence of control subjects, largely due to the high prevalence of OSA in the stroke sample. Additionally, genetic testing for APOE4 and other risk factors was not conducted, which limits the completeness of the risk factor assessment for PSCI.

## 6 Conclusions

Early CPAP therapy in stroke patients could contribute to improved cognitive performance. The observed decline in pNFL levels supports ongoing neuronal recovery in the subacute phase of stroke and does not show a neurodegenerative process involved in PSCI in our study. However, a direct relationship with OSA and its treatment remains inconclusive. Advanced age, history of prior stroke, and anxiety symptoms emerged as significant contributors to poorer cognitive outcomes.

Given the substantial burden of PSCI on individuals and healthcare systems, our results emphasize the importance of early screening for OSA, personalized therapeutic approaches, and interventions aimed at improving CPAP adherence. The high prevalence of OSA in our cohort, juxtaposed with the complete absence of prior OSA diagnosis or treatment, highlights significant gaps in screening and possibly systemic or country-specific barriers to care, warranting further investigation.

## CRediT authorship contribution statement

**Petra Levicka:** Writing – original draft, Investigation, Data curation. **Miriam Slavkovska:** Investigation. **Dominik Koren:** Investigation. **Joaquim Ventosa:** Investigation. **Ján Hlodak:** Validation, Software, Formal analysis, Data curation. **Jana Papikova:** Validation, Project administration, Data curation. **Zuzana Gdovinova:** Writing – review & editing, Methodology, Conceptualization. **Eva Feketeova:** Writing – review & editing, Supervision, Methodology, Funding acquisition.

## Funding

This work was supported by Vedecká grantová agentúra MŠVVaŠ SR a SAV grant No. 1/0439/24.

## Declaration of competing interest

The authors declare that they have no known competing financial interests or personal relationships that could have appeared to influence the work reported in this paper.
